# Brevilin A Induces Cell Cycle Arrest and Apoptosis in Nasopharyngeal Carcinoma

**DOI:** 10.3389/fphar.2019.00594

**Published:** 2019-05-24

**Authors:** Rui Liu, Zhao Qu, Yushan Lin, Chi-Sing Lee, William Chi-Shing Tai, Sibao Chen

**Affiliations:** ^1^State Key Laboratory of Chinese Medicine and Molecular Pharmacology (Incubation), The Hong Kong Polytechnic University Shenzhen Research Institute, Shenzhen, China; ^2^Department of Chemistry, The Hong Kong Baptist University, Hong Kong, China; ^3^Department of Applied Biology and Chemical Technology, Hong Kong Polytechnic University, Hong Kong, China; ^4^Institute of Medicinal Plant Development, Chinese Academy of Medical Sciences and Peking Union Medical College, Beijing, China

**Keywords:** brevilin A, nasopharyngeal carcinoma, anti-cancer, cell cycle arrest, apoptosis induction

## Abstract

Nasopharyngeal carcinoma (NPC) is one of the most common malignant cancers in Southeast Asia and Southern China. *Centipeda minima* extract (CME) had previously demonstrated anti-cancer effects in human NPC. Brevilin A, a sesquiterpene lactone isolated from *C. minima*, has been reported to exhibit biological activities. In this study, we investigated its anti-NPC effect and further explored its molecular mechanisms. The effects of brevilin A were tested in the NPC cell lines CNE-1, CNE-2, SUNE-1, HONE1, and C666-1. Effects of brevilin A on cell viability were determined by MTT assay, and cell cycle and apoptosis were detected by flow cytometry. The molecular mechanism of cell cycle regulation and apoptosis were investigated via Western blot. Results showed that brevilin A inhibited NPC cell viability in a concentration- and time-dependent manner. Brevilin A induced cell cycle arrest at G2/M and induced apoptosis. Western blot results demonstrated that brevilin A could down-regulate cyclin D3, cdc2, p-PI3K, p-AKT, p-mTOR, and p-STAT3, while up-regulating cleaved PARP, cleaved caspase 9, and Bax. Regulation of cyclin B1, cdk6, and Bcl-2 expression by brevilin A showed dynamic changes according to dose and time. In the tumor xenograft model, brevilin A could reduce tumor growth, at a similar magnitude to cisplatin. However, notably, whereas cisplatin treatment led to significant weight loss in treated mice, treatment with brevilin A did not, indicating its relative lack of toxicity. Taken together, brevilin A regulated cell cycle, activated the caspase signaling pathway, and inhibited PI3K/AKT/mTOR and STAT3 signaling pathways *in vitro*, and exhibited similar efficacy to the common chemotherapeutic cisplatin *in vivo*, without its associated toxicity. These findings provide a framework for the preclinical development of brevilin A as a chemotherapeutic for NPC.

## Introduction

Nasopharyngeal carcinoma (NPC) is a type of head and neck cancer and is one of the most common malignant cancers in Southeast Asia and especially Southern China (Wei et al., 2010; Dou et al., 2014; Liang et al., 2016). In China, NPC incidence and mortality, respectively, account for 38.29% and 40.14% of global figures (1.2/100,000; 0.7/100,000) (Wei et al., 2010; Liang et al., 2016). Further, incidence and mortality of NPC are substantially higher in men than in women, and also adolescents are more likely to suffer from NPC, while the elderly, especially those over 65 years of age, exhibit relatively higher incidence and mortality rates. The main causal factors of NPC include genetic susceptibility, dietary factors, Epstein–Barr virus (EBV) infection, smoking, alcohol, and workplace exposure (Ozoya et al., 2016; Turati et al., 2017). Presently, the standard primary treatment for NPC is radiotherapy, but this may cause brain radiation injury (Li et al., 2013). More and more studies have focused on traditional Chinese medicines (TCM), which have significant efficacy, but with relatively minor toxic side effects (Mazzio et al., 2016).

**Figure 1:**
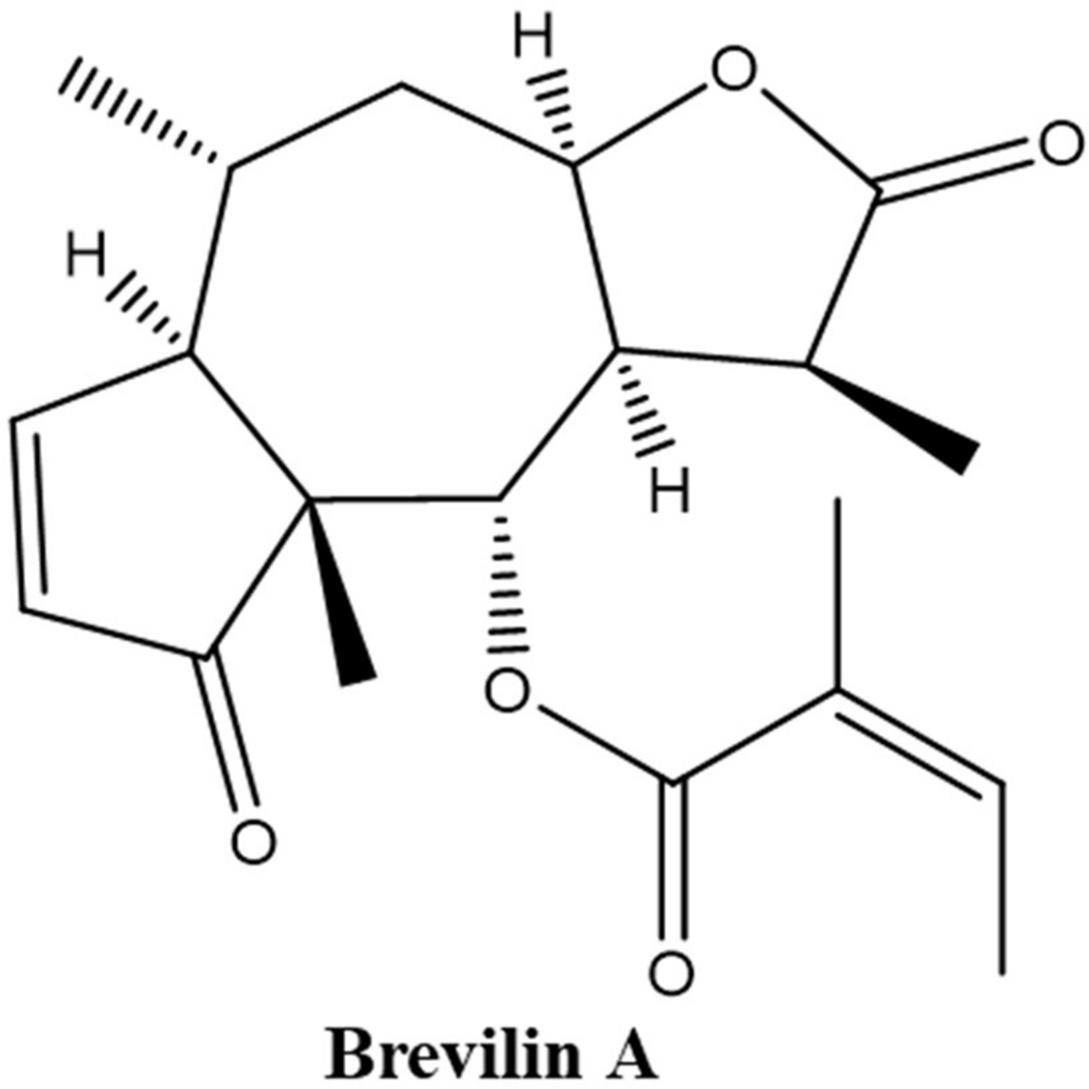
Chemical structure of brevilin A.

Centipedae herba, the dried whole plant of *Centipeda minima* (L.) A.Braun & Asch. (Ebushicao) (Asteraceae) (CM), has been used as a TCM for several thousands of years, and is commonly used for the treatment of rhinitis, sinusitis, pain, swelling (State Pharmacopoeia Commission of People’s Republic of China, 2015; Mazzio et al., 2016), cancer (Chen et al., 2011; Guo et al., 2013; Guo et al., 2015), etc. CM contains flavones and their glycosides, phenolic and polyphenolic acids, and sesquiterpene lactones (SQLs) (Liu et al., 2005; Chan et al., 2016). SQLs are built from three isoprene units, contain one or more lactone rings, and exhibit the obvious anti-cancer (Wang et al., 2017), anti-oxidant (Shoaib et al., 2017), and anti-inflammatory (Scarponi et al., 2014) activities.

(3*S*,3a*R*,4*S*,4a*R*,7a*R*,8*R*,9a*R*)-3,4a,8-Trimethyl-2,5-dioxo-2,3,3a,4,4a,5,7a,8,9,9a-decahydroazuleno[6,5-b]furan-4-yl (2*Z*)-2-methyl-2-butenoate, also named 6-*O*-angeloylplenolin, brevilin A, or brevifolin (Liu et al., 2005; Ding et al., 2009; Chan et al., 2016; Li et al., 2018) (**Figure 1**), is an SQL isolated from CM and has been reported to exhibit biological activities, including anti-bacterial (Taylor and Towers, 1998) and anti-cancer (Chen et al., 2013; Liu et al., 2013) effects. The *in vitro* anti-cancer effect of brevilin A has been demonstrated in a multitude of different cancers, including human multiple myeloma, breast cancer, lung cancer, and colon carcinoma (Li et al., 2008; Liu et al., 2011; Su et al., 2011; Chen et al., 2013; Liu et al., 2013; Liu et al., 2015; Cheng et al., 2017; Wang et al., 2017; Wang et al., 2018; You et al., 2018).

However, to date, there has been only one study on the *in vitro* effects of brevilin A against NPC (Su et al., 2011). In this study, the authors found that brevilin A could inhibit proliferation, regulate the cell cycle, and induce apoptosis in human NPC cells. Thus, based on previous research, where CM extract demonstrated anti-cancer effects in NPC (Guo et al., 2013; Guo et al., 2015), in the present study, the anti-cancer mechanisms of its isolated compound, brevilin A, in NPC were further investigated.

## Materials and Methods

### Drugs and Reagents

Brevilin A, at a purity of 98%, was purchased from Jiangsu Yongjian Pharmaceutical Co., Ltd. (Jiangsu, China). Cisplatin was obtained from the National Institutes for Food and Drug Control (Beijing, China). Dulbecco’s Modified Eagle’s Medium (DMEM), Dulbecco’s phosphate-buffered saline (DPBS), fetal bovine serum (FBS), penicillin streptomycin, and 0.25% trypsin–EDTA were purchased from Gibco (Gibco Life Technologies, Grand Island, NY, USA). Thiazolyl blue tetrazolium bromide (MTT), dimethyl sulfoxide (DMSO), and bovine serum albumin were purchased from Sigma (St. Louis, MO). Annexin V-FITC cell apoptosis detection kit and Cell cycle detection kit were obtained from Beyotime Institute of Biotechnology (Beyotime, Shanghai, China). The Pierce bicinchoninic acid (BCA) protein assay kit was obtained from Thermo Scientific Inc. (Rockford, IL, USA).

Skim milk, TBS buffer premixed powder, and Western blotting substrate were obtained from Solarbio Life Sciences and Sangon Biotech. Primary antibodies PI3K, AKT, mTOR, Bcl-1, Bax, cyclin B1, cyclin D3, and CDK6, and secondary antibodies were purchased from Cell Signaling Technology Inc. (Beverly, MA, USA). β-Actin primary antibody was purchased from Zsbio Commerce Store (Beijing, China). Tanon High-sig ECL Western Blotting Substrate kit was purchased from Tanon Science & Technology Co., Ltd. (Shanghai, China).

### Cell Culture and Drug Treatments

Human NPC cell lines CNE-1, CNE-2, SUNE-1, HONE1, and C666-1 were supplied by the Hong Kong NPC AoE Cell Line Repository.

CNE-1, CNE-2, SUNE-1, and HONE1 cells were grown in DMEM with 10% heat-inactivated FBS and 1% penicillin/streptomycin. C666-1 cells were grown in Roswell Park Memorial Institute medium (RPMI) 1640 with 10% FBS and 1% penicillin/streptomycin. All cells were maintained in an incubator with a humidified atmosphere and 5% CO_2_ at 37°C.

### Cell Viability Assay

The cytotoxic effects of brevilin A on NPC cells were assessed using the MTT cell viability assay (Mei et al., 2007; Guo et al., 2015). Inhibition of cell proliferation was measured by the reduction of 3-(4,5-dimethylthiazol-2-yl)-2,5-diphenyltetrazolium bromide (MTT) to formazan. Cells were seeded (2,000 cells/well for CNE-1, CNE-2, SUNE-1; 4,000 cells/well for HONE1; and 15,000 cells/well for C666-1) in 96-well plates. After overnight incubation, cells were treated with varying concentrations of brevilin A for 24, 48, or 72 h (0–50 µM). MTT reagent was then added to the medium in each well and incubated for 4 h at 37°C. Reduced formazan crystals were solubilized in DMSO and optical density values were measured at 570 nm on a micro plate reader. The proliferation inhibition rates of brevilin A were calculated using the following equation:

$$Inhibition(\%)=\left[1-\left(OD_{drug}-OD_{blank}\right)\;/\;\left(OD_{control}-OD_{blank}\right)\right]\times 100$$

Six parallel wells were set for each group. The average value was obtained with three-time repeated tests. The half-maximal inhibitory concentration (IC_50_) values were calculated using GraphPad Prism software (version 5.0).

### Cell Cycle Analysis

The effects of brevilin A on the cell cycle were detected by flow cytometry (Wang et al., 2015). Briefly, following 24 or 48 h treatment with 0–10 µM brevilin A, cells were harvested. Ice-cold 70% ethanol was added to fix the cells at 4°C overnight. After that, cells were rinsed with ice-cold PBS and incubated with 25 µl of propidium iodide and 10 µl of RNase for 30 min at 37°C in the dark. Flow cytometric cell analysis was performed using a BD Accuri C6 flow cytometry system (Becton Dickson Immunocytometry-Systems, San Jose, CA, USA). CellQuest software (ModFit software, Verity Software House, Inc., Topsham, ME, USA) was used to determine the proportions of cells in different cell stages of cell cycle progression (G0/G1, S, and G2/M phases). DNA content distribution was analyzed using ModFitLT software.

### Annexin V-FITC Apoptosis Assay

According to the manufacturer’s introductions, the Annexin V-FITC Apoptosis Detection kit was employed for double staining with FITC-Annexin V and PI (Luo et al., 2010). Briefly, cells seeded in 60-mm dishes were treated with 0–50 µM BA and then collected after 24 and 48 h. After the addition of 195 µl of binding buffer, the cells were incubated with 5 µl of FITC-labeled Annexin V and 10 µl of PI at room temperature for 20 min in the dark. The samples were then placed on ice in the dark and assessed by FACS analysis. Cells were discriminated into viable, necrotic, early apoptotic, and late apoptotic cells after analysis by flow cytometry.

### Morphological Observation

Cells were seeded onto cell culture dishes for attachment and then treated by different concentrations of brevilin A for 24 and 48 h.

To visualize DNA, the cells were washed with PBS, fixed for more than 30 min in 75% ethanol, stained with 4′,6′-diamidino-2-phenylindole hydrochloride (DAPI) for 10 min at room temperature in the dark, and then rinsed with PBS twice. The stained cells were visualized under a fluorescence microscope (Olympus, Tokyo, Japan) with excitation and emission wavelengths of 350 and 460 nm, respectively.

### Western Blot Analysis

After treatment with different concentrations of brevilin A for 24 and 48 h, respectively, cells were collected for Western blot analysis. Ice-cold RIPA buffer with protease and phosphatase inhibitors was used for cell lysis. Cell lysates were then centrifuged at 14,000 × *g* for 15 min at 4°C to pellet cell debris, and the supernatants were transferred to a new tube. The protein content of the samples was measured using the Pierce BCA Protein Assay kit (Thermo Scientific, USA).

Protein samples were prepared in 5× loading buffer with heating at 98°C for 5 min. Proteins were separated by sodium dodecyl sulfate polyacrylamide gel electrophoresis (SDS-PAGE) with 8% or 12% polyacrylamide gels in a Bio-Rad Mini-Protean apparatus (Bio-Rad Laboratories, Inc., USA) followed by transfer to PVDF membranes by wet transfer. After transfer, membranes were washed with TBST, and blocked in 5% skim milk diluted in TBST. Membranes was washed three times for 5 min each with 10 ml of TBST. After that, the membrane was incubated with primary antibody (1:1,000) in 5% BSA with gentle agitation overnight at 4°C. After washing three times, the membrane was incubated with horseradish peroxidase (HRP)-linked secondary antibody (Cell Signaling Technology, Beverly, MA, USA) (1:2,000) in 5% BSA with gentle agitation for 2 h at room temperature, and washed three times for 5 min each with 15 ml of TBST.

The signals were detected by Tanon High-sig ECL Western Blotting Substrate kit according to the procedures of the manufacturer’s specifications. Finally, the band of the protein signals was quantified by area using ImageJ and calculated according to the reference bands of β-actin.

### Mouse Xenograft Tumor Model

Male BALB/c nude mice were purchased from Beijing Vital River Laboratory Animal Technology Co., Ltd. (Beijing China) and were kept in an specific pathogen free (SPF)-grade animal facility, where temperature is within the range of 22 ± 2°C and the humidity ranged from 40% to 70%, under a 12-h light/dark cycle. Food and water were available *ad libitum*, and mice were examined daily. Mice were subcutaneously injected in their right flanks with 2 × 10^6^ CNE-2 cells suspended in 100 μl of PBS. After a 9-day inoculation, when tumors grew to an average volume of 110 mm^3^, mice were randomly arranged into different experimental groups. In the vehicle control group, mice were treated with 0.5% carboxymethyl cellulose (CMC). In the brevilin A treatment groups, mice were orally fed daily with 10 or 20 mg/kg brevilin A in 0.5% CMC. In the positive control group, mice were injected intraperitoneally with 5 mg/kg cisplatin two times weekly. Tumor sizes were measured every 2 days and calculated using the formula (length × width^2^)/2 mm^3^. After the 16-day experimental period, mice were killed, and their tumors and vital organs were harvested and weighed. Tumors were snap frozen in liquid nitrogen and stored at −80°C. Upon use, tumors were lysed in RIPA buffer and submitted to Western blot analysis as above. All animal experiments were approved by the Hong Kong Polytechnic University Animal Subjects Ethics Subcommittee and conducted in accordance with the Institutional Guidelines and Animal Ordinance of the Department of Health.

### Statistical Analysis

Statistical analysis was calculated by one-way analysis of variance (ANOVA) by GraphPad Prism 5.0 software (GraphPad Software, Inc., La Jolla, CA, USA). All data are expressed as means ± standard deviation (SD) (*in vitro* experiments) or standard error of the mean (SEM) (*in vivo* experiments) of no less than three different replicates. The criterion for statistical significance was *p* < 0.05.

## Results

### Brevilin A Inhibited the Proliferation of NPC Cells* In Vitro*

**Figure 2:**
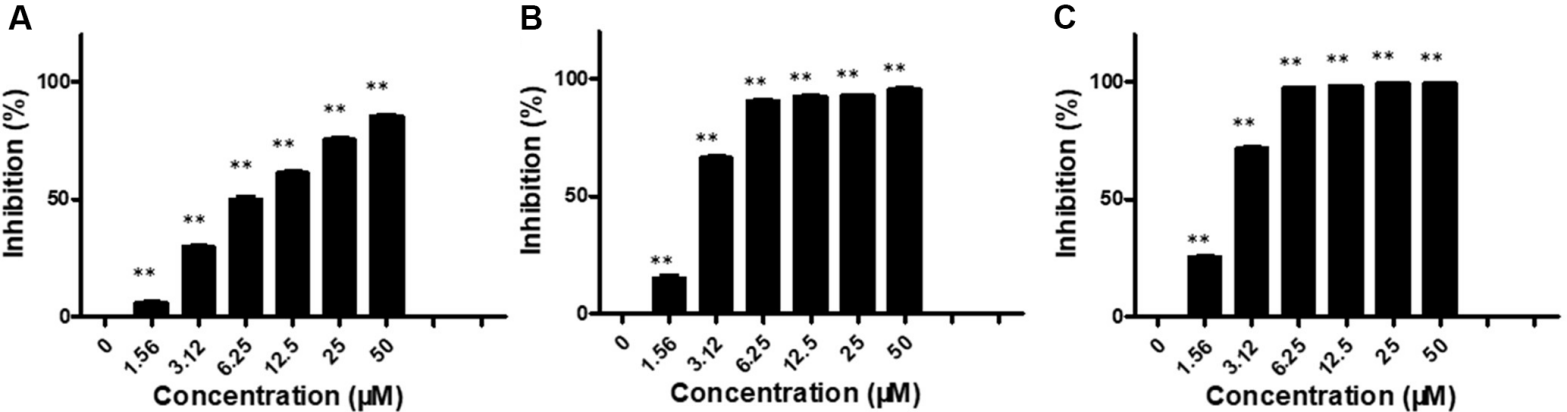
Effects of brevilin A on proliferation of CNE-2 cells. CNE-2 cells were treated with different concentrations (0–50 μM) of brevilin A for **(A)** 24 h, **(B)** 48 h, and **(C)** 72 h, after which MTT assay was used to evaluate its anti-proliferative effects. Cells without drug treatment were used as a control. Data are shown as mean ± SD. **p < 0.01, compared with control.

We first evaluated the *in vitro* anti-proliferation activity of brevilin A on NPC cells (CNE-1, CNE-2, SUNE-1, HONE1, and C666-1 cells). Cells were treated with various concentrations (0–50 μM) of brevilin A for 24, 48, and 72 h. Results from the MTT assay showed that brevilin A exerted a cytotoxic effect on the cells, significantly inhibiting the growth of a variety of NPC cells, including CNE-1, CNE-2, SUNE-1, HONE1, and C666-1, in a dose- and time-dependent manner (**Figure 2**, **Supplementary Figure S1**, **Supplementary Table 1**). For CNE-2 cells, after treatment with brevilin A (1.56, 3.12, 6.25, 12.50, 25.00, and 50 μM) for 24 h, inhibition was 5.83, 29.55, 49.74, 61.04, 75.20, and 85.34%, respectively (**Figure 2**). Similar to results at 24 h, the inhibitory rates were increased with increasing concentrations of brevilin A at 48 and 72 h. Brevilin A of 3.125 µM inhibited cell growth by 29.55% at 24 h, 66.24% at 48 h, and 71.63% at 72 h. Obviously, the inhibitory rates of brevilin A on CNE-2 cells were also increased with time. The calculated IC_50_ values of brevilin A in CNE-2 cells with treatment times of 24, 48, and 72 h were 7.93, 2.60, and 22.26 µM, respectively. Analogously, the inhibitory trend of brevilin A in other NPC cells was increased with dose and time.

### Cell Morphology

**Figure 3:**
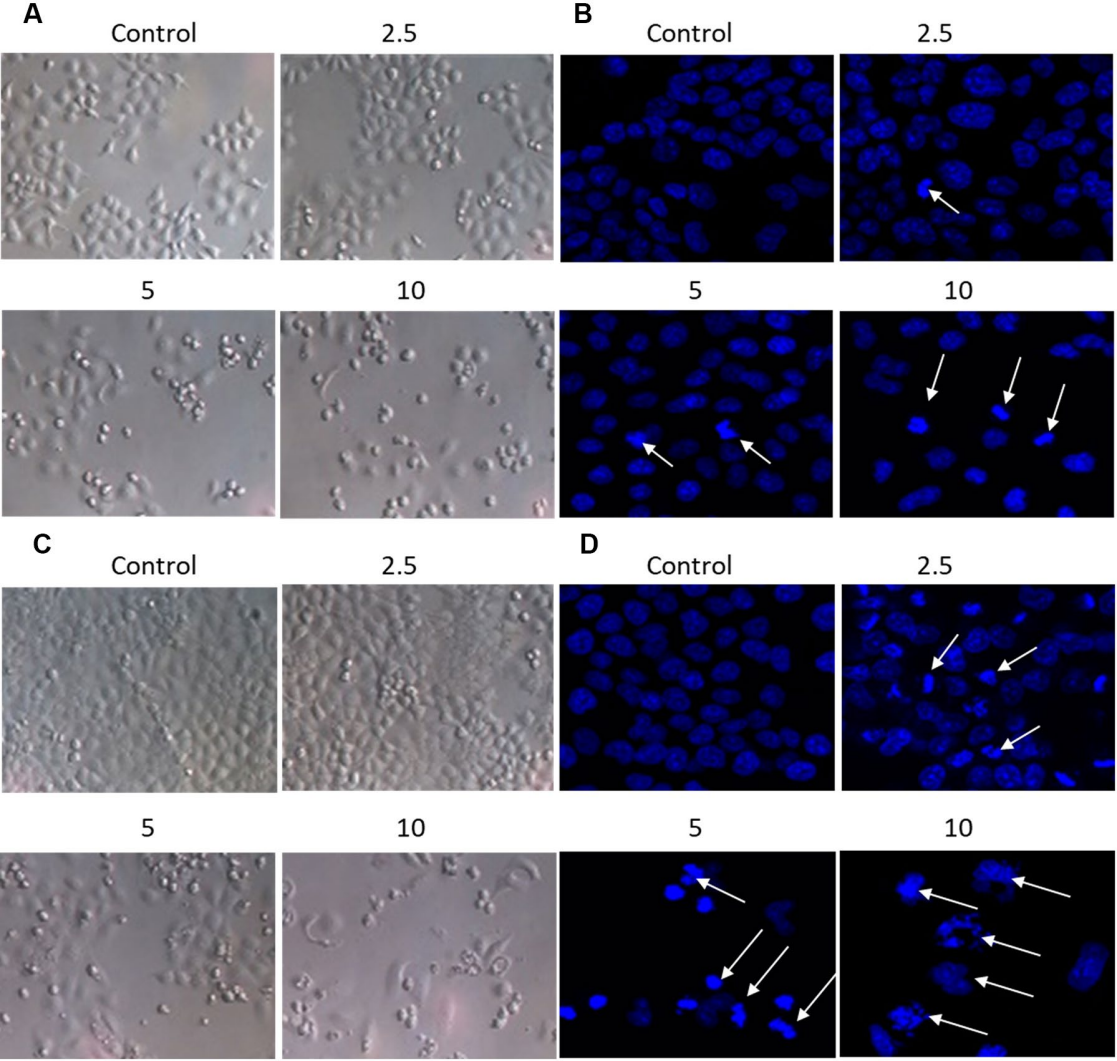
Morphological changes of CNE-2 induced by brevilin A. CNE-2 cells were treated with different concentrations of brevilin A (2.5–10 μM) for **(A** and **B)** 24 h, or **(C** and **D)** 48 h, and then cell morphology was observed under an optical microscope (magnification, 100×). Cells were then stained with DAPI, and their nuclear morphologies were observed using confocal microscopy (magnification, 200×). Arrows indicate hallmarks of apoptosis, including chromatin concentration and formation of apoptotic bodies.

The effect of brevilin A on the morphology of NPC cells was directly observed under an inverted microscope, or observed via laser confocal microscopy after DAPI staining (**Figure 3A–D**). After treatment of CNE-2 cells with various doses of brevilin A, nuclear morphological changes were observed after DAPI staining. Cells of control group (24 and 48 h) showed normal cell architecture with distinct cytoskeletons, while cells of brevilin A treatment groups exhibited typical morphological changes including cell shrinkage, high chromatin condensation, visible formation of apoptotic bodies and nuclear degradation, which associated with apoptosis (**Figure 3B and D**). This phenomenon indicated that brevilin A induced apoptosis besides growth inhibition on NPC cells in a dose-dependent manner.

### Brevilin A Modulated Cell Cycle Progression of NPC Cells

In order to determine the effect of brevilin A on cell cycle progression of NPC cells, the distribution of cells at different stages was analyzed by flow cytometry.

**Figure 4:**
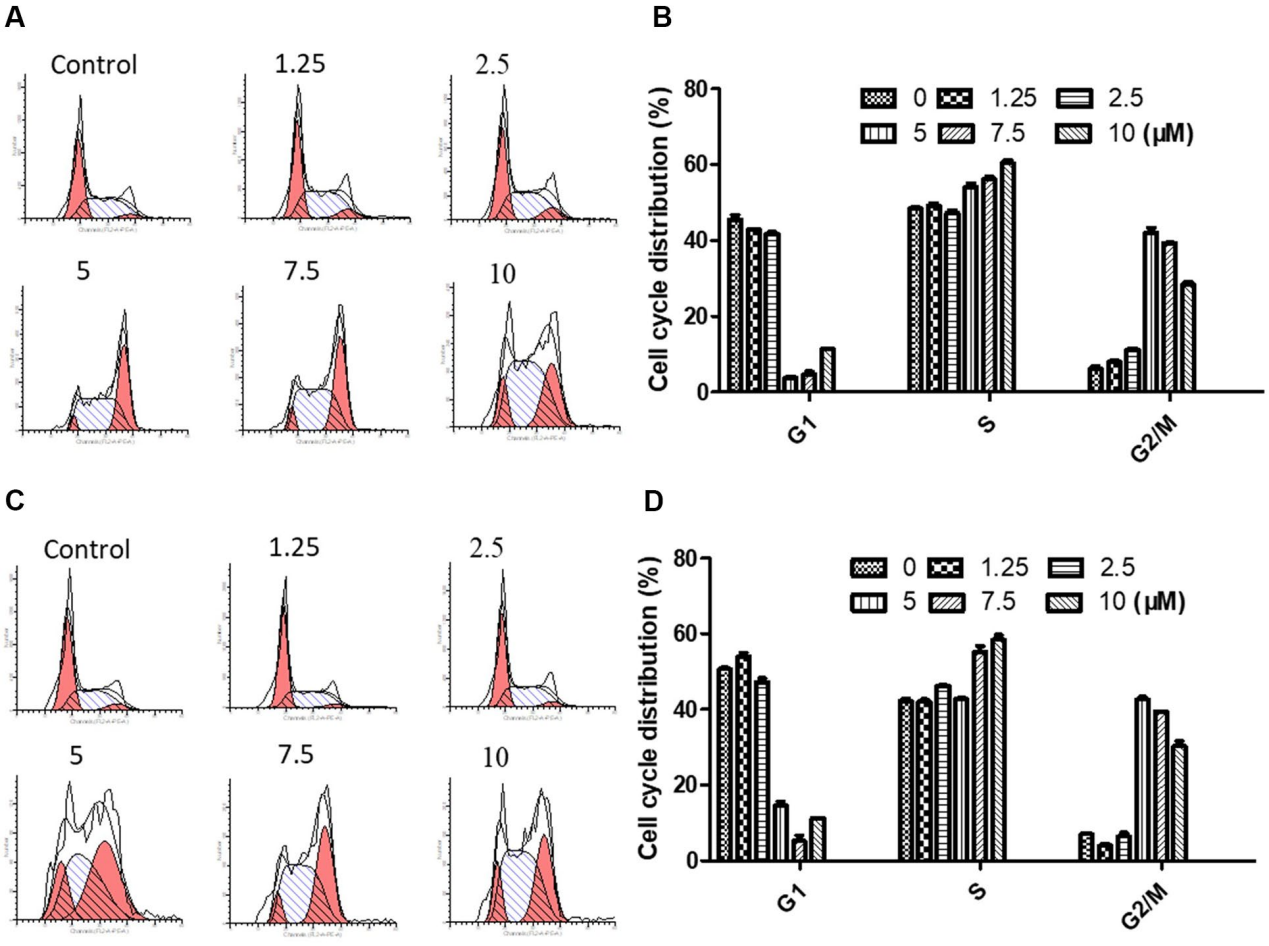
Brevilin A induced cell cycle arrest at the G2/M phase in CNE-2 cells. CNE-2 cells were treated with brevilin A at 1.25 to 10 μM for 24 and 48 h and then detected by flow cytometry. Representative DNA fluorescence histograms of PI-stain cells showed the cell cycle distribution for **(A)** 24 h and **(C)** 48 h. Quantitative bar graphs of the proportion of CNE-2 cells in different phases for after 24 h **(B)** and 48 h **(D)** treatment. Values are shown as the means ± SD and the representative of three independent experiments (*n* = 3).

NPC cells were treated to brevilin A at concentrations of 1.25, 2.5, 5, 7.5, and 10 µM for 24 h and 48 h, and analyzed. Flow cytometry results showed that at 24 and 48 h, CNE-2 cells were arrested at G2/M by brevilin A at concentrations ranging from 5 to 10 µM (**Figure 4A–D**). Cell arrest at G2/M was dramatically increased in a dose-dependent manner, and the percentage of cells in the G2/M phase reached its highest point at 5 µM (approximately 40% at both 24 and 48 h), while the percentages of those in the control group were 5.42% at 24 h and 7.29% at 48 h, respectively. No significant variations in the percentages of cells in S phase were observed after brevilin A treatment. Meanwhile, it was correspondingly accompanied by a significant decrease in G1 phase compared to the control cells without treatment of brevilin A. Taken together, these results exhibited the ability of cell cycle regulation of brevilin A in NPC cells, which may be associated with proliferation and apoptosis.

### Flow Cytometric Analysis of Apoptosis

Annexin V-FITC/PI double staining was carried out to detect apoptosis by flow cytometry. The results showed that NPC cells treated with brevilin A exhibited a significant increase in apoptotic cell populations (early + late) compared to control without treatment in a dose- and time-dependent manner.

**Figure 5:**
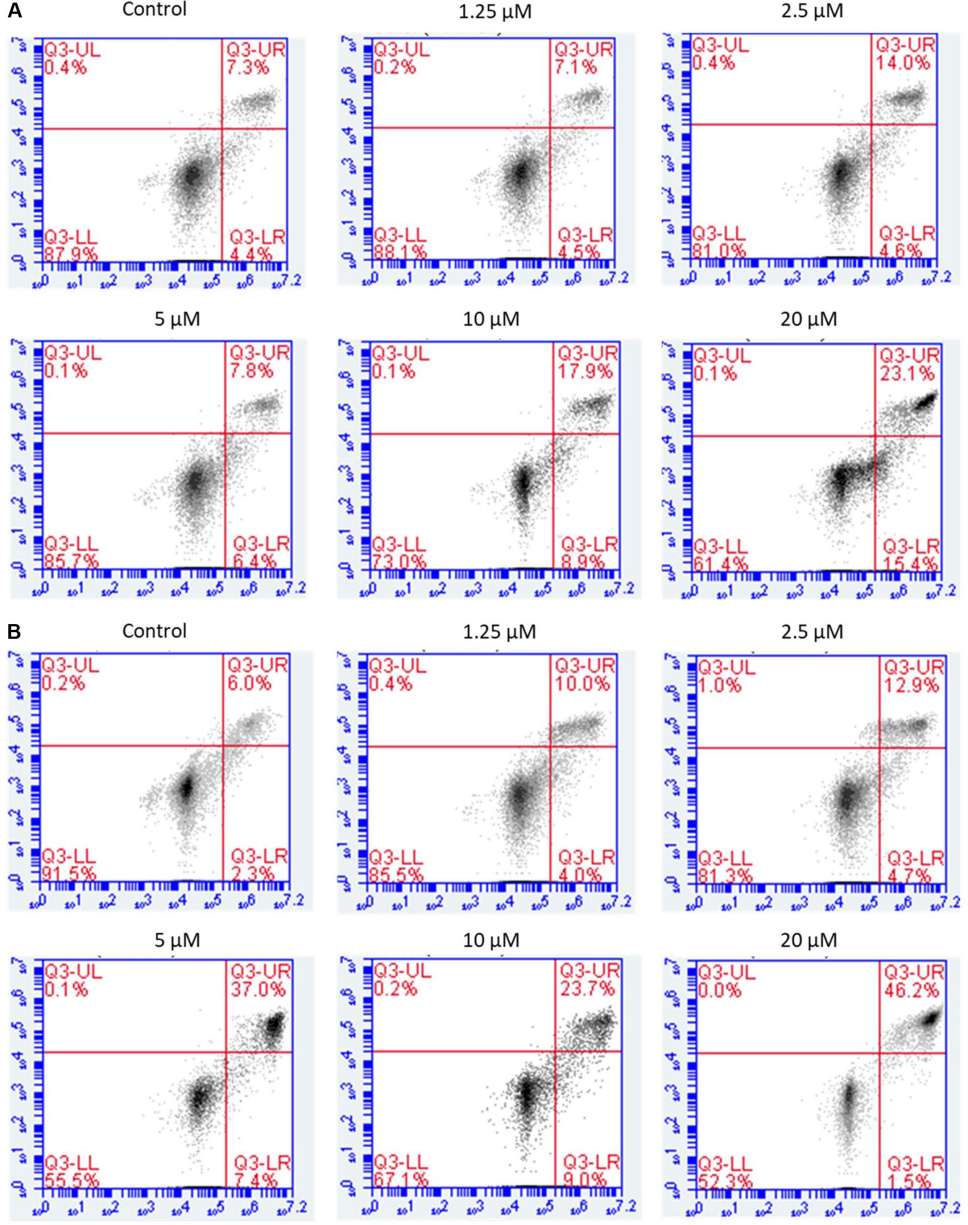
Flow cytometric analysis of induction of apoptosis by brevilin A in CNE-2 cells. CNE-2 were cells treated with brevilin A at 1.25 to 10 μM for 24 and 48 h, stained using a commercially available Annexin-V/PI kit and analyzed by flow cytometry. Representative images showing the cells after 24 h **(A)** and 48 h **(B)** treatment. Quantification of apoptotic cell populations are shown, including early apoptotic (bottom right quarter) and late apoptotic cells (top right quarter).

For CNE-2 cells treated with brevilin A (1.25, 2.5, 5, 7.5, 10, 20, or 50 µM) for 24 h, percentage of apoptotic cells ranged from 11.6% to 61.9% (**Figure 5A**). At 48 h, the percentage of apoptotic cells ranged from 8.3% to 69.1% (**Figure 5B**). For example, after treatment with 5 µM brevilin A for 24 and 48 h, the percentage of apoptotic cells were 14.2% and 44.4%, respectively, while that of control groups was only approximately 10%. Strikingly, the results clearly suggested that brevilin A induced cell apoptosis of NPC cells.

### Western Blot Analysis of Cell-Cycle-Related Proteins

**Figure 6:**
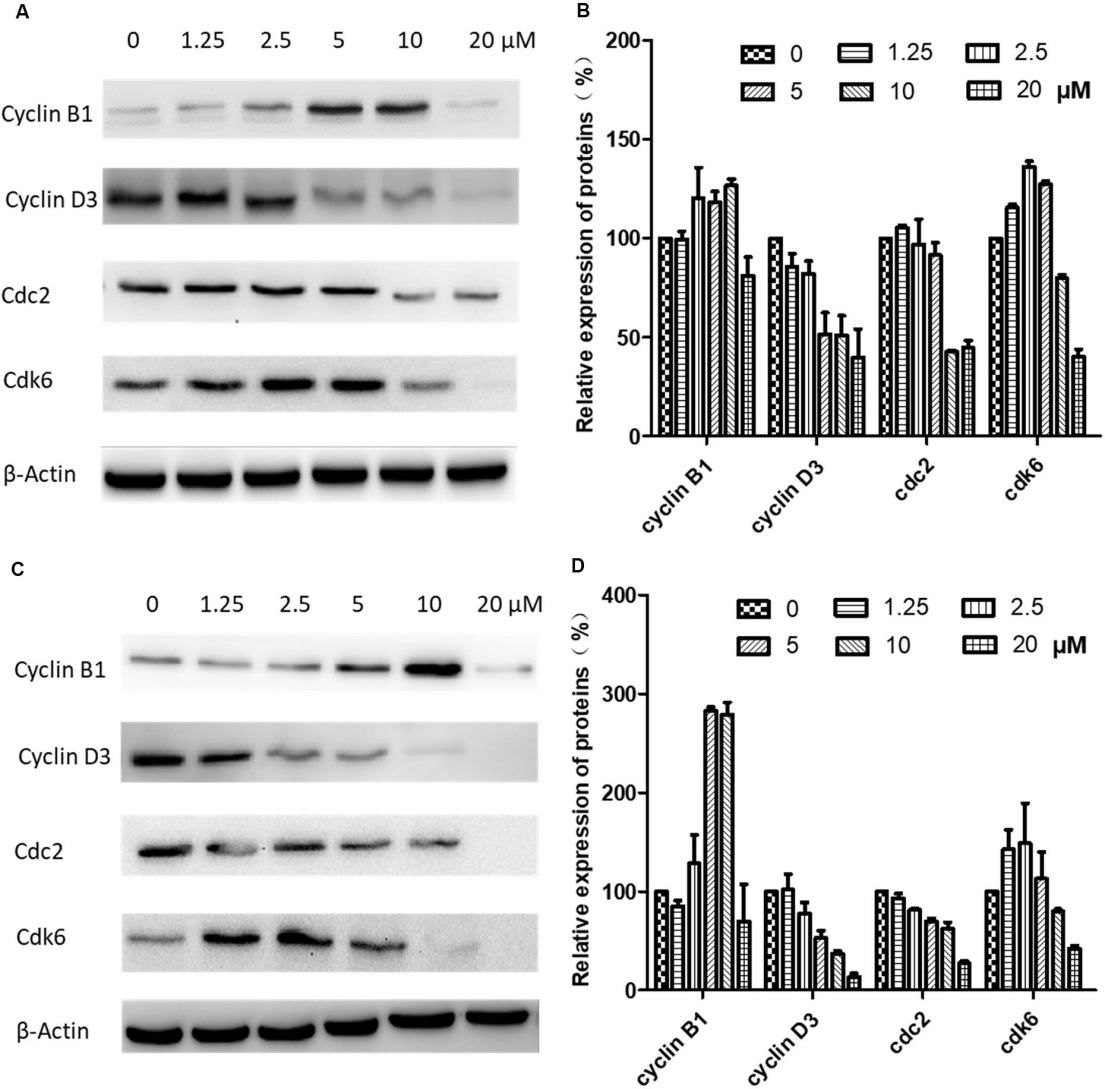
Effects of brevilin A on expression of key cell cycle proteins in CNE-2 cells. CNE-2 cells were treated with brevilin A at concentrations of 1.25–20 μM for 24 and 48 h, and cell lysates were harvested and subjected to Western blot analysis using antibodies against cyclin B1, cyclin D3, cdc2, and cdk6. β-Actin was used as an internal control. Results of Western blot are shown for **(A)** 24-h and **(C)** 48-h treatment. Bar graphs **(B** and **D)** quantifying the relative expression of proteins. Data are expressed as mean ± SD.

Western blotting was carried out to examine the expression levels of key regulators responsible for the G2/M checkpoint including cyclin B1, cyclin D3, cdk1/cdc2, and cdk6. As shown in **Figure 6**, in CNE-2 cells treated with 0–20 µM brevilin A for 24 h (**Figure 6A and B**) and 48 h (**Figure 6C and D**), the expression levels of cyclin D3 and cdk1/cdc2 were decreased in comparison with control cells. In addition, the expression levels of cyclin B1 and cdk6 were respectively dose-dependently increased and significantly decreased, compared with control cells.

### Expression of Apoptosis-Related Proteins

**Figure 7:**
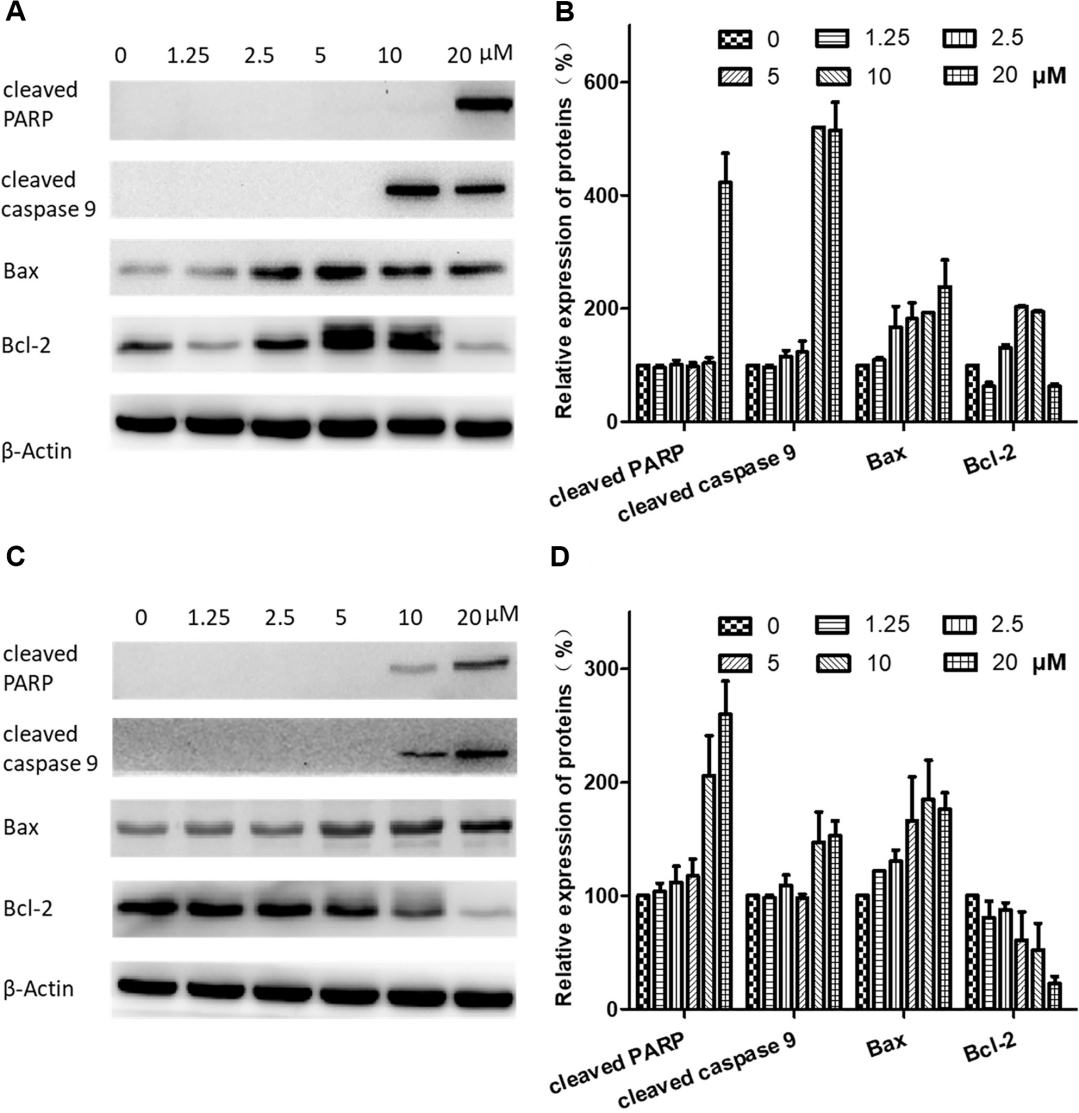
Effects of brevilin A on expression of apoptotic proteins in CNE-2 cells. CNE-2 cells were treated with brevilin A at concentrations of 1.25–20 μM for 24 and 48 h, and cell lysates were harvested and subjected to Western blot analysis using antibodies against cleaved PARP, cleaved caspase 9, bax, and bcl-2. β-Actin was used as an internal control. Results of Western blot are shown for **(A)** 24-h and **(C)** 48-h treatment. Bar graphs **(B** and **D)** quantifying the relative expression of proteins. Data were expressed as mean ± SD.

To further investigate the mechanisms of brevilin A-induced apoptosis in NPC cells, the expression of apoptosis-related proteins including cleaved poly(ADP-ribose) polymerase (cleaved PARP) (p85), cleaved caspase 9, bax, and bcl-2 was evaluated by Western blot. After treatment with brevilin A (1.25, 2.5, 5, 7.5, 10, or 20 µM) for 24 h (**Figure 7A and B**) or 48 h (**Figure 7C and D**), levels of cleaved PARP (p85), cleaved caspase 9, and bax were dramatically increased (10 or 20 µM). Interestingly, at 24 h, the expression of the anti-apoptotic protein bcl-2 first increased and then decreased upon treatment with increasing concentrations of brevilin A, while at 48 h, bcl-2 was down-regulated in a dose-dependent manner.

### Regulation of the PI3K/AKT and STAT3 Signaling Pathways

**Figure 8:**
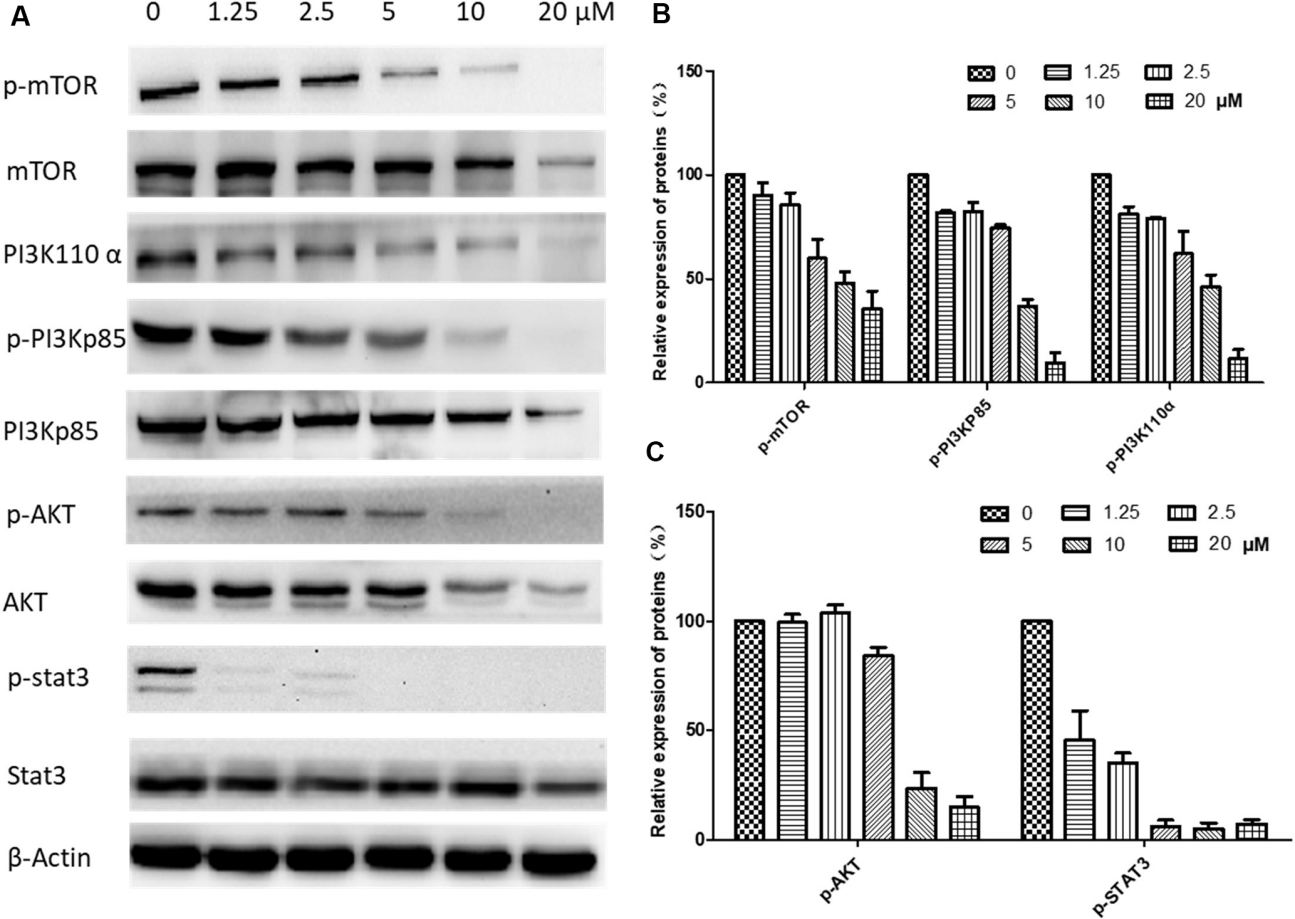
Effects of brevilin A on regulation of PI3K/AKT pathway members in CNE-2 cell (48 h). CNE-2 cells were treated with brevilin A at concentrations of 1.25–20 μM for 48 h, and cell lysates were harvested and subjected to Western blot analysis using antibodies against mTOR and p-mTOR (Ser^2481^), PI3K p110 α, PI3K p85, and p-PI3K p85 (Tyr^458^), AKT and p-AKT (Ser^473^), and STAT3 and p-STAT3 (Tyr^705^). β-Actin was used as the internal control. **(A)** Results of Western blot after 48-h treatment. Bar graphs **(B** and **C)** quantifying the relative expression of proteins. Data are expressed as mean ± SD.

The underlying mechanisms for brevilin A-induced apoptosis were then further explored. We assessed whether brevilin A treatment down-regulated the expression levels of proteins in the PI3K–Akt–mTOR pathway. After treatment with brevilin A at various concentrations for 24 h (**Supplementary Figure 2**) and 48 h (**Figure 8**), the levels of PI3K p110α, p-PI3K p85, p-Akt, and p-mTOR were markedly decreased in a concentration-dependent manner compared to control. Investigation of the effects of brevilin A on STAT3 signaling showed that the compound could inhibit the protein expression of p-STAT3 in CNE-2 cells. To confirm the involvement of brevilin A in the above pathways in NPC, we repeated our tests in the HONE1 cell line. Similarly, in HONE1, brevilin A treatment also down-regulated expression levels of PI3K–Akt–mTOR and STAT3 pathway proteins after 24-h (**Supplementary Figure 3**) and 48-h (**Supplementary Figure 4**) treatments.

### Brevilin A Inhibited CNE-2 Xenograft Tumor Growth *In Vivo*

**Figure 9:**
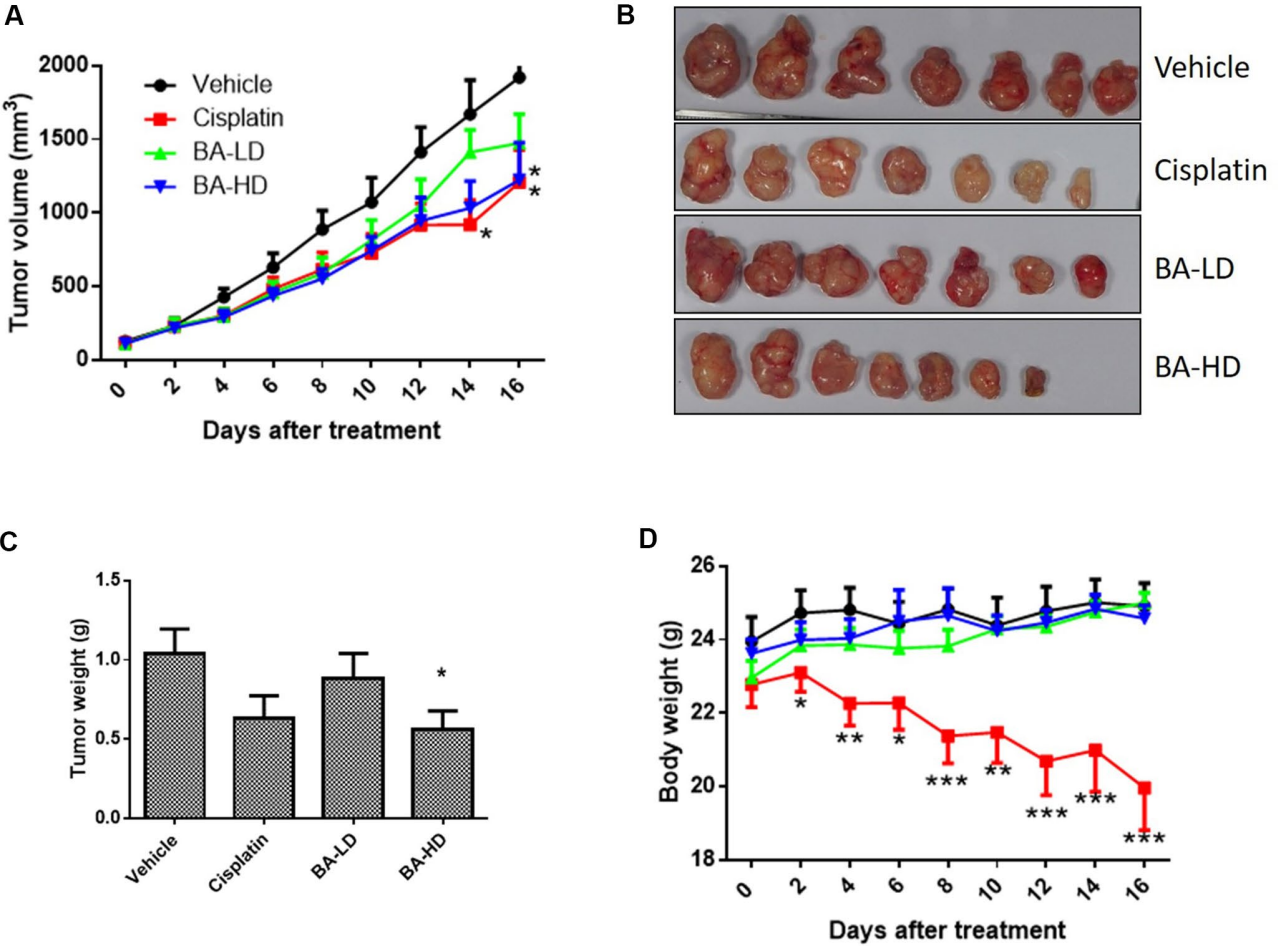
*In vivo* anti-cancer effects of brevilin A in CNE-2 xenograft nude mice. CNE-2 xenograft nude mice received treatment with brevilin A at low (10 mg/kg) or high (20 mg/kg) doses daily via oral gavage, or cisplatin (5 mg/kg) twice weekly via i.p. injection. A vehicle control group received daily oral gavage of 0.5% CMC. The treatment period lasted for 16 days, after which mice were sacrificed and tumors were excised and weighed. **(A)** Average tumor volumes throughout the 16-day experimental period. **(B)** Representative photograph of tumors at experimental endpoint. **(C)** Average tumor weight at experimental endpoint. **(D)** Mouse body weight throughout the 16-day experimental period. Data are expressed as mean ± SEM.

To study the *in vivo* anti-cancer effects of brevilin A, we employed a CNE-2 xenograft tumor mouse model. In this experiment, mice received oral treatment of high-dose (20 mg/kg) or low-dose (10 mg/kg) brevilin A, or i.p. injection of cisplatin as a positive control, for 16 days. At the experimental endpoint, in mice treated with 20 mg/kg brevilin A, average tumor volumes and weights were significantly (*p* < 0.05) decreased by 36.3% and 46.0%, respectively, when compared to vehicle control, while the anti-tumor efficacy of cisplatin was 37.1% and 39.3%, respectively (**Figure 9A–C**). While cisplatin treatment led to a significant decrease in the body weight of mice by 19.9% (*p* < 0.001), brevilin A did not cause any significant body weight loss in mice (**Figure 9D**). These results indicate the improved anti-cancer efficacy and reduced toxicity of brevilin A when compared to the commonly used chemotherapeutic, cisplatin.

### Brevilin A Inhibited PI3K/AKT and STAT3 Signaling *In Vivo*

**Figure 10:**
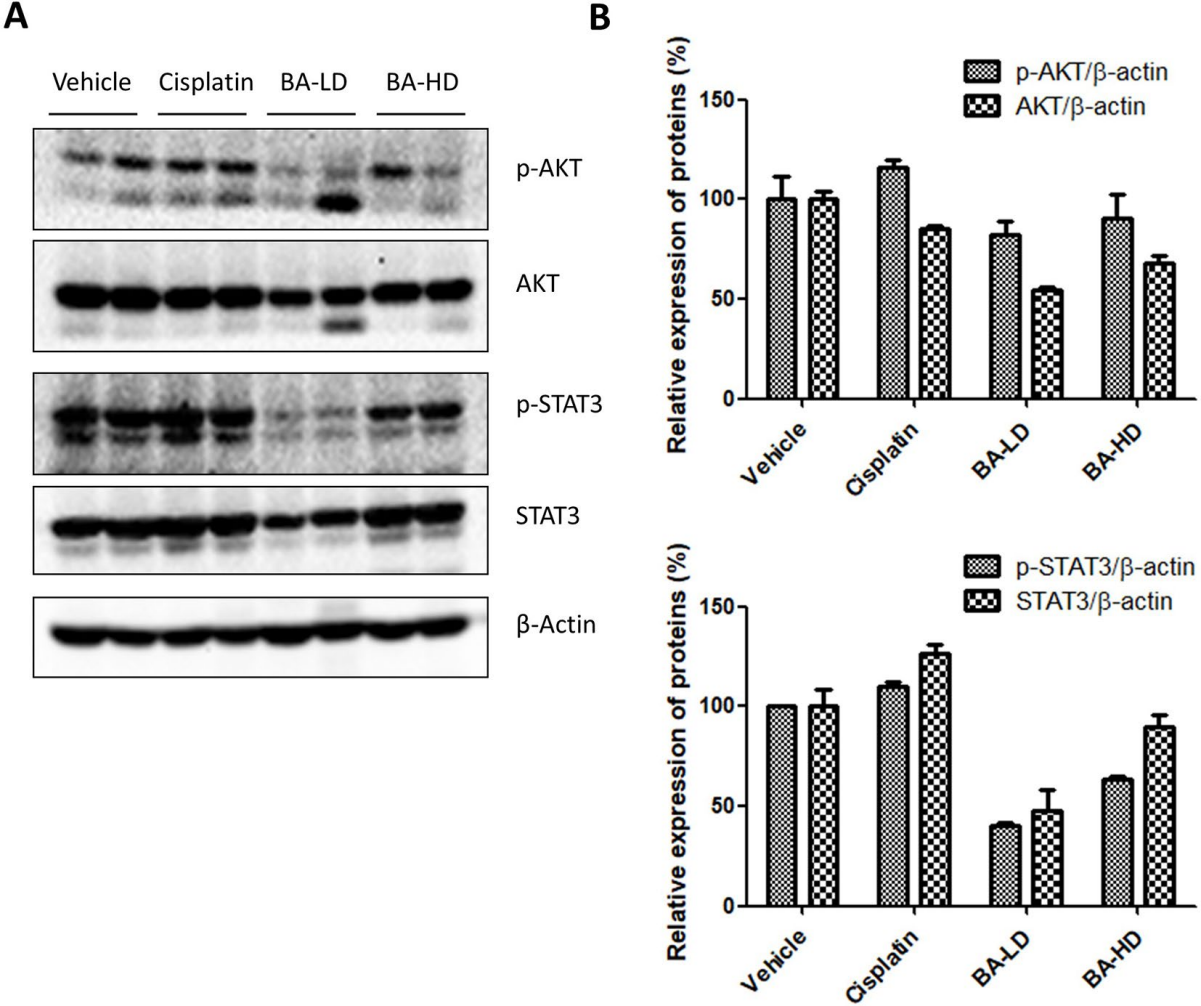
Effect of brevilin A on the PI3K/AKT and STAT3 signaling pathways of CNE-2 xenograft tumors. **(A)** At the end of a 16-day treatment period, tumors from brevilin A-treated CNE-2 xenograft nude mice were excised and lysed for Western blot analysis using antibodies against AKT and p-AKT (Ser^473^) and STAT3 and p-STAT3 (Tyr^705^). β-Actin was used as an internal control. **(B)** Quantifications of the relative expression of proteins. Data are expressed as mean ± SD.

To examine whether our *in vitro* findings on the involvement of BA on PI3K/AKT and STAT3 signaling would also hold true *in vivo*, we investigated the protein expression of key members of the two signaling pathways in tumor lysates from brevilin-A-treated mice. Results showed that brevilin A at both low and high doses could inhibit the protein expression of p-AKT and p-STAT3, the activated forms of the proteins (**Figure 10**), echoing our results from the *in vitro* studies.

## Discussion

NPC is an endemic cancer, which has obvious inclination towards racial and geographic distribution. NPC is much more common in certain parts of North Africa and Asia, particularly Southeast China (Cao et al., 2014; Oncology, 2017). The high incidence of NPC in Southern China and Hong Kong has led to it also being referred to as “Guangdong Cancer” or “Guangdong Tumor” (Wei et al., 2010; Cao et al., 2014; Liang et al., 2016). Patients with this high-mortality and high-morbidity cancer not only bear heavy economic pressure, but also great psychological pressures. Increasingly, TCM has been reported in the prevention and treatment of NPC, which can provide an alternative to chemotherapy and its associated side effects.

*C. minima* is a TCM usually used for treatment of rhinitis by herbalist doctors in China. Most modern research shows that *C. minima* possesses anti-bacterial, anti-inflammatory, and anti-cancer effects (Chen et al., 2011; Mazzio et al., 2016). In previous studies, volatile oils extracted from *C. minima* were found to have anti-NPC activity in the CNE cell line, via induction of the intrinsic pathway of apoptotic cell death (Su et al., 2010). Additionally, we have previously shown that *C. minima* ethanol extract (CME) could inhibit the proliferation of CNE-1 cells and activation of the PI3K–AKT–mTOR signaling pathway (Guo et al., 2015). Brevilin A is a single compound belonging to the SQL class of chemicals isolated from *C. minima*, with a variety of pharmacological activities (Liu et al., 2005; Chan et al., 2016; Bosco and Golsteyn, 2017). Previous studies have demonstrated the anti-cancer activity of SQLs isolated from *C. minima* in various cancers, including colon cancer (Huang et al., 2014), prostate cancer (Vishwanath Pradeep et al., 2012), and NPC (Su et al., 2009). In addition, SQLs from *C. minima* have been demonstrated to exert anti-angiogenic effects, which may potentially contribute to its anti-cancer activity (Huang et al., 2016). However, prior to this study, the anti-cancer effects of brevilin A in NPC have not yet been investigated.

The present study demonstrated that brevilin A inhibited the viability of NPC cells in a time- and dose-dependent manner by MTT assay. Treatment with brevilin A led to significant changes in cell morphology, including cell shrinkage and vacuolization. DAPI staining presented sapphirine nuclei with lobular, fragmented shapes and fringe collection, consistent with the induction of apoptosis.

The cell cycle is a complex and elaborate process, and alterations in its modulation may result in abnormal metabolism and proliferation. To investigate whether brevilin A could affect the cell cycle of NPC cells, cell cycle progression was analyzed by flow cytometry with or without treatment of brevilin A. Brevilin A markedly induced G2/M phase arrest in NPC cells in a dose- and time-dependent manner, which was consistent with the previously published findings (Su et al., 2011). As G2/M is critical to cell cycle progression, the molecular mechanism of cell cycle regulation by brevilin A was further investigated. Cyclin B1 plays an important role in the G2/M transition as well as in M phase progression. The expression of cyclin B1 protein is increased in the G2 phase and rapidly decreased during anaphase, which may be associated with inhibition of mitosis (King et al., 1994; Widrow et al., 1997). Brevilin A up-regulated expression of cyclin B1 at concentrations ranging from 1.25 to 10 µM, while inhibiting its expression at 20 µM, which may be out of the range for cell cycle regulation and instead induce apoptosis. Previous studies have shown that the expression of cyclin B1 after brevilin A treatment was altered depending on its dose and time (Liu et al., 2011; Liu et al., 2015). Also, increased expression of cyclin B1 was shown to result in G2/M arrest (Liu et al., 2011). Overexpression of cdc2 results in over-proliferation and causes tumor formation. Cdc2 interacts with cyclin B1 and, in the late stage of G2, forms the maturation promoting factor (MPF), which is a key protein in regulation of mitosis. In the present study, the protein expression of cdc2 was reduced after brevilin A treatment, implying that cdc2 participated in the brevilin-A-induced G2/M arrest. Moreover, brevilin A decreased the expression of cyclin D3 and regulated the expression of cdk6. This is in line with previous studies, which also showed that brevilin A could inhibit the expression of cyclin D3 (Su et al., 2011).

Apoptotic induction in cancer cells is a crucial therapeutic strategy for cancer treatment. After treatment of brevilin A, morphological changes of the NPC cells demonstrated hallmarks of apoptosis. This induction of apoptosis was confirmed by flow cytometry using an annexin V-FITC/PI double-staining approach. The number of early and late apoptotic NPC cells was increased, in brevilin-A-treated cells compared to control. The results suggested that brevilin A was able to induce NPC cell apoptosis. Our findings are consistent with those in previous studies, where brevilin A was shown to induce apoptosis in cancer cells (Wang et al., 2017; Wang et al., 2018; You et al., 2018).

Expression of apoptosis-related proteins was then evaluated by Western blotting to investigate the mechanisms of brevilin-A-induced apoptosis. Apoptosis ultimately leads to a change in the expression of cleaved caspases and brevilin A has previously been demonstrated to participate in the modulation of caspases in human multiple myeloma (Liu et al., 2013). After treatment with brevilin A for 24 or 48 h, the expression of cleaved caspase 9 in CNE-2 cells was increased. Expression of cleaved PARP (p85) was also increased compared with control cells, altogether indicating that induction of the caspase apoptotic pathway may be one mechanism for brevilin-A-induced cell apoptosis. This is consistent with previous articles, which showed that cleaved caspase 9 and cleaved PARP were found in apoptotic cells after treatment with brevilin A (Lin et al., 2017; Wang et al., 2018).

The B cell lymphoma 2 (Bcl-2) family of proteins includes death antagonists such as Bcl-2 and Bcl-xl, and death agonists such as Bax and Bad, which play an important role in the regulation of mitochondrial-mediated apoptosis. Increased expression of Bcl-2 has been observed in a variety of human cancer cells and tissues, where it can suppress a number of apoptotic death programs. Brevilin A was previously reported to induce mitochondrial apoptosis in U87 glioblastoma cells and induce increased expression of cleaved caspase 9 and cleaved PARP (Wang et al., 2018). In this study, brevilin A down-regulated the expression of Bcl-2 and up-regulated the expression of Bax at 48 h, resulting in apoptosis in CNE-2 cells. Interestingly, the expression of Bcl-2 in our present study was first increased and then decreased in CNE-2 cells with treatment of brevilin A for 24 h. This may potentially be due to the involvement of Bcl-2 multiple signaling pathways in addition to the apoptotic pathway, raising a valuable research issue, and the potential to identify new targets for treatment of NPC.

Proliferation and apoptosis of cancers are regulated by multi-signaling pathways, such as PI3K/AKT/mTOR, MAPK/ERK, and STAT3 (Daniel and Mcleod, 2005; Mei et al., 2007; Gao and Tian, 2010; Guo et al., 2015; Cheng et al., 2017). The activation or inhibition of these signaling pathways is not isolated but interrelated and interactive with each other, each being a point in a network of relations. The PI3K/AKT/mTOR pathway is essential for regulating cell survival and apoptosis and is frequently found mutated and/or overactivated in human cancers (Liu et al., 2014). It has been reported that brevilin A inhibits the PI3K/AKT/mTOR pathway to induce apoptosis and autophagy in many cancers (Guo et al., 2013; You et al., 2018). In this study, our results demonstrate that brevilin A suppresses PI3K/AKT/mTOR activity in CNE-2 cells and in *in vivo* CNE-2 tumor xenografts, which may be associated with inhibition of NPC cell proliferation and induction of apoptosis.

In addition, the expression of STAT3 was investigated in the study. Levels of STAT3 in NPC tissues have been shown to be higher than that in normal tissues, exhibiting a negative correlation with survival rates (Cheng et al., 2018). Brevilin A has been reported to decrease the expression of p-STAT3 in many types of cancers such as lung, breast, and liver (Chen et al., 2013; Cheng et al., 2017). In the same vein, in this study, we found that brevilin A could inhibit the expression of p-STAT3 in CNE-2 cells. This inhibitory effect may contribute to induction of apoptosis and suppression of cancer cell migration (Cheng et al., 2017). Inhibition of STAT3 activation can also lead to the inhibition of AKT activation (Chen et al., 2013), which also reflected our *in vitro* and *in vivo* findings, indicating multiple potential pathways for the anti-cancer activity of brevilin A.

Lastly, in our *in vivo* studies, we found that at a dose of 20 mg/kg, brevilin A exhibited similar anti-cancer efficacy to the commonly used chemotherapeutic cisplatin, but without the associated toxicity that accompanies the chemo drug. This indicates the potential of brevilin A as an alternative to traditional chemotherapeutics, with comparable anti-cancer activity, but reduced amounts of side effects, providing patients with additional options for treatment to NPC.

## Conclusion

Taken together, in this study, we have shown that brevilin A could inhibit cell proliferation and induce apoptosis in human NPC cell lines. The mechanisms of brevilin A may be involved in induction of cell cycle arrest at the G2/M phase, activating the PI3K/AKT/mTOR signaling pathway and the mitochondrial apoptosis pathway. The anti-proliferative and apoptosis-inducing anti-cancer activity of brevilin A in NPC likely depends on interactions between members of a network of signaling pathways. In animal studies, we found that brevilin A exhibited similar anti-cancer efficacy to cisplatin, but without the associated toxicity. Our findings have demonstrated the anti-NPC effect of brevilin A and offer solid groundwork for further preclinical research of its activity in NPC.

## Data Availability Statement

The raw data supporting the conclusions of this manuscript will be made available by the authors, without undue reservation, to any qualified researcher. 

## Ethics Statement

All animal experiments were approved by the Hong Kong Polytechnic University Animal Subjects Ethics Sub-committee and conducted in accordance with the Institutional Guidelines and Animal Ordinance of the Department of Health.

## Author Contributions

C-SL, WC-ST, and SC contributed to conception and design of the study. RL, ZQ, and YL performed all the experiments. RL and ZQ wrote the manuscript. WC-ST and SC contributed to manuscript revision. All authors read and approved the submitted version.

## Funding

This work was co-supported by the Basic Research Foundation of Shenzhen Science and Technology Innovation Committee (JCYJ20151030164022389 and JCYJ20160229173844278), the National Natural Science Foundation of China (No. 81872769), and China Postdoctoral Science Foundation (Grant No. 2018M640827).

## Conflict of Interest Statement

The authors declare that the research was conducted in the absence of any commercial or financial relationships that could be construed as a potential conflict of interest. The handling editor declared a shared affiliation, though no other collaboration, with several of the authors at the time of review.
