# Supplementary material for: Brevilin A Induces Cell Cycle Arrest and Apoptosis in Nasopharyngeal Carcinoma
Source: Front Pharmacol. 2019 May 24;10:594. doi: 10.3389/fphar.2019.00594 (PMC6544084; doi:10.3389/fphar.2019.00594)
Supplement: Supplementary file 1 [file DataSheet_1.docx]

Supplementary Material

**Supplementary Table 1.** Inhibitory effects of brevilin A on proliferation of human NPC cells.

|  | IC_50_ (μM) | | |
| --- | --- | --- | --- |
| Cell line | 24 h | 48 h | 72 h |
| CNE-2 | 7.93 | 2.60 | 2.26 |
| C666-1 | 6.17 | 4.12 | 3.52 |
| CNE-1 | 5.60 | 2.56 | 2.47 |
| SUNE-1 | 8.74 | 3.01 | 1.80 |
| HONE1 | 9.58 | 2.26 | 1.46 |


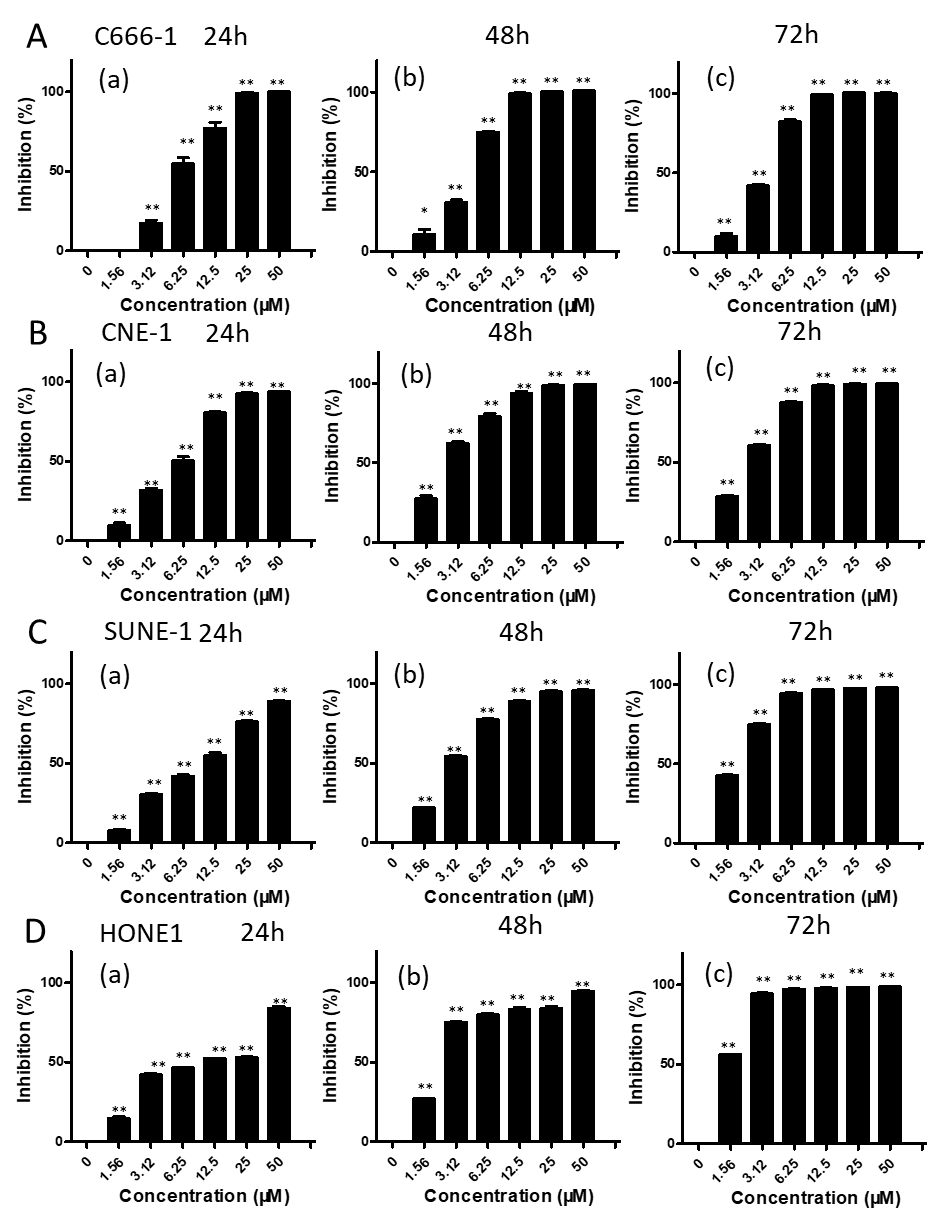


**Supplementary Figure 1.** Effects of Brevilin A on anti-proliferation of human NPC cells. (**A**) C666-1, (**B**) CNE-1, (**C**) SUNE-1, and (**D**) HONE1 cells were treated with different concentrations (0-50 μM) of brevilin A for 24 h, 48 h, and 72 h, after which MTT assay was used to evaluate their anti-proliferation effects. Cells without drug treatment were used as a control. Data are shown as mean ± SD. * *p* ＜ 0.05, ** *p* ＜ 0.01, compared with control.


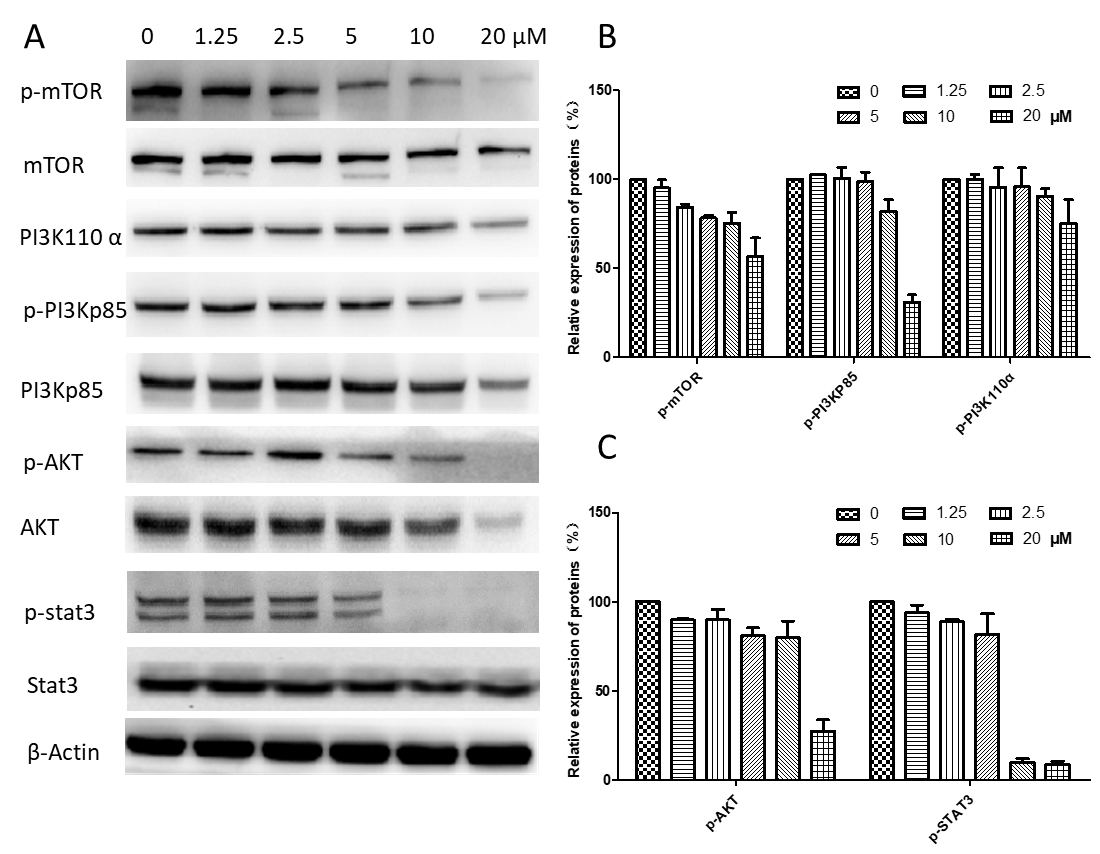


**Supplementary Figure 2.** Regulation of PI3K/AKT pathway by brevilin A in CNE-2 (24 h). CNE-2 cells were treated with brevilin A at concentrations of 1.25-20 μM for 24 h, and cell lysates were harvested and subjected to Western blot analysis using antibodies against mTOR and p- mTOR (Ser^2481^), PI3K p110 α, PI3K p85 and p- PI3K p85 (Tyr^458^), AKT and p- AKT (Ser^473^), STAT3 and p-STAT3 (Tyr^705^). β-actin was used as the internal control. Results of Western blot are shown in (**A**). (**B and C**) Quantitative bar graphs showing the relative expression of phospho-proteins, by calculating the ratio relative to total proteins. Data are expressed as mean ± SD.


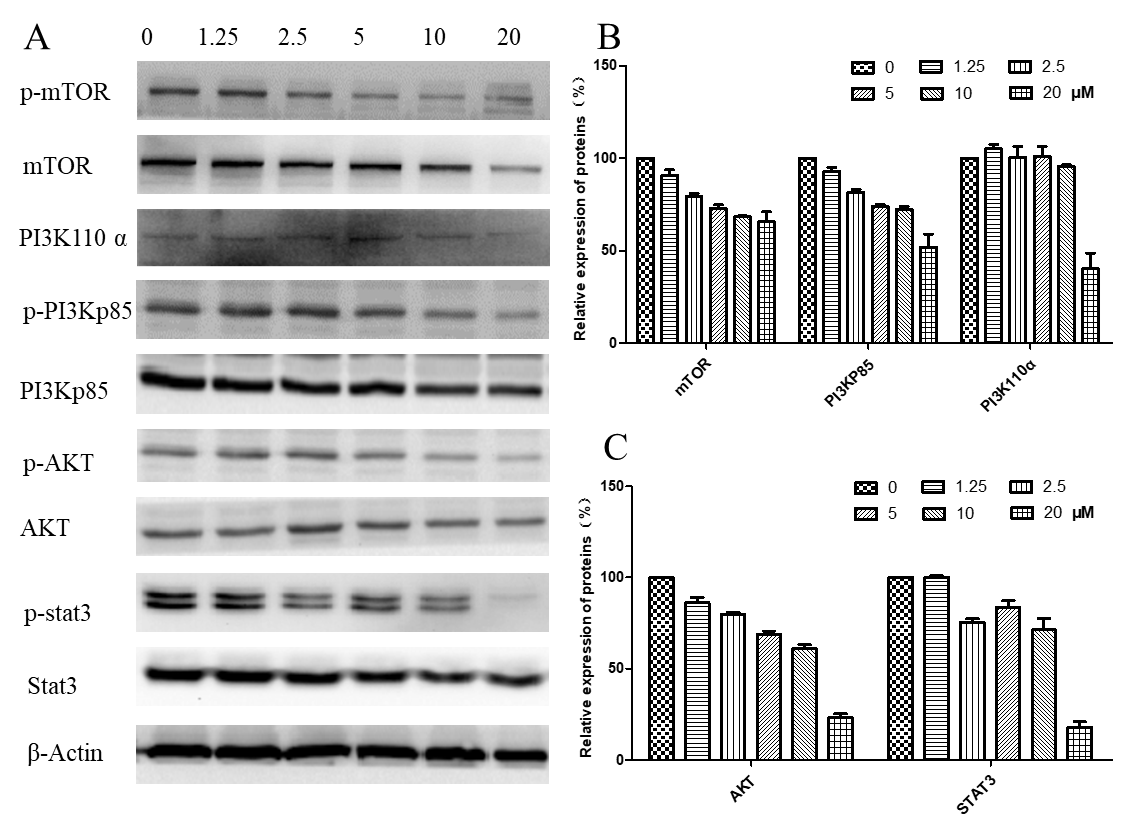


**Supplementary Figure 3.** Regulation of the PI3K/AKT pathway by brevilin A in HONE1 cells (24 h). HONE1 cells were treated with brevilin A at concentrations from 1.25-20 μM for 24 h. Cell lysates were harvested and subjected to Western blot analysis using antibodies against mTOR, p‑mTOR (Ser^2481^), PI3K p110 α, PI3K p85 and p-PI3K p85 (Tyr^458^), AKT, p-AKT (Ser^473^), STAT3 and p-STAT3 (Tyr^705^). β-actin was used as an internal control. Results are shown in (**A**). (**B and C**) Bar graphs showing the relative expression of phosphorylated proteins relative to total proteins. Data are expressed as mean ± SD.


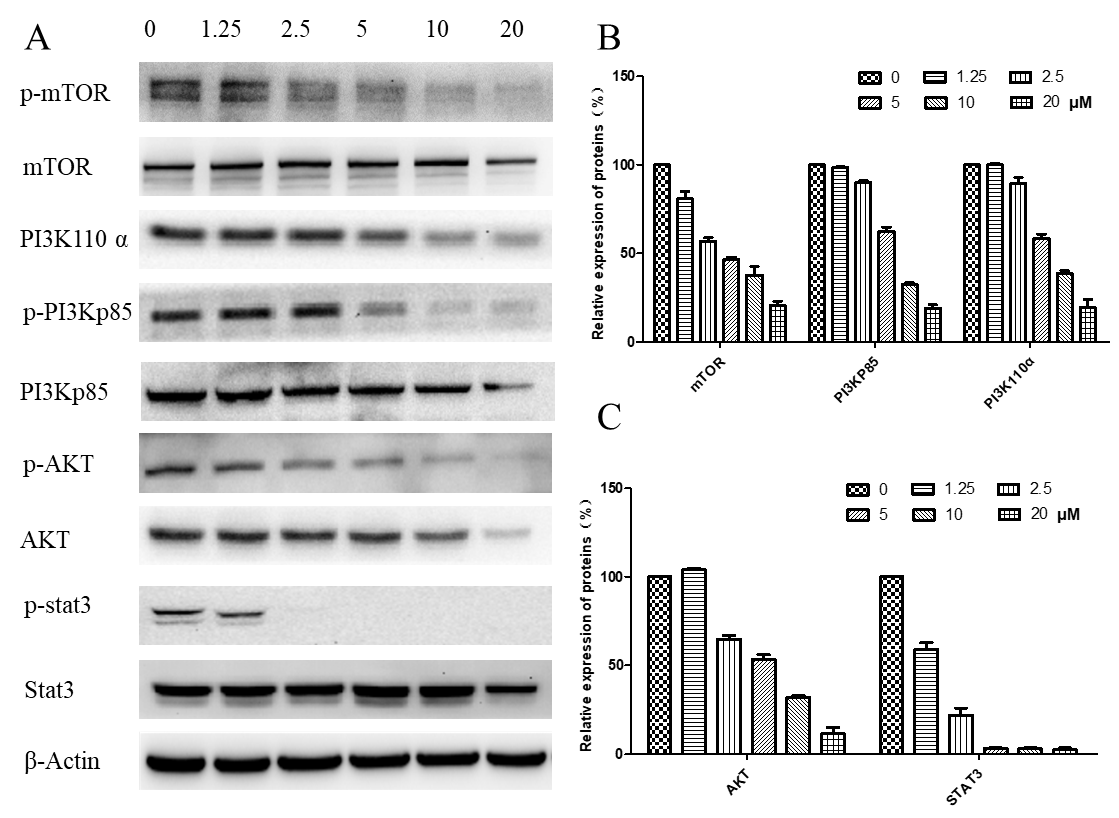


**Supplementary Figure 4. Regulation of the PI3K/AKT pathway by brevilin A in HONE1 cells (48 h).** HONE1 cells were treated with brevilin A at concentrations from 1.25-20 μM for 48 h. Cell lysates were harvested and subjected to Western blot analysis using antibodies against mTOR, p‑mTOR (Ser^2481^), PI3K p110 α, PI3K p85 and p-PI3K p85 (Tyr^458^), AKT, p-AKT (Ser^473^), STAT3 and p-STAT3 (Tyr^705^). β-actin was used as an internal control. Results are shown in (**A**). (**B and C**) Bar graphs showing the relative expression of phosphorylated proteins relative to total proteins. Data are expressed as mean ± SD.
